# Nonsurgical Treatment of Type II Dens Invaginatus in a Maxillary Lateral Incisor Using Cone-Beam Computed Tomography 

**DOI:** 10.22037/iej.v13i1.19091

**Published:** 2018

**Authors:** Mohsen Afkar, Mahboubeh Gholamshahi, Mehdi Mohammadi

**Affiliations:** a * Department of Endodontics, Dental Branch of Tehran, Islamic Azad University, Tehran, Iran; *; b * Dentistry Student, Dental Branch of Tehran, Islamic Azad University, Tehran, Iran*

**Keywords:** Cone-Beam Computed Tomography, Dens Invaginatus, Maxillary Lateral Incisor

## Abstract

This is a clinical report of a case of Oehlers type II dens invagination in left maxillary lateral incisor. A 12-year-old female patient was referred to endodontic department of Islamic Azad University. She reported history of pain and swelling on left anterior maxilla. Due to the insufficient information from conventional radiography, cone-beam computed tomography (CBCT) was ordered. CBCT revealed apical lucency and two separate canals. Conventional root canal therapy was done using warm vertical technique for invaginated canal. One year follow-up radiographies showed periapical repair and absence of symptoms.

## Introduction

Dens invagination (DI) or dens in dente has been defined as an anomaly resulting from uncontrolled growth of amelodental structure into the dental papillae before the calcification stage [1, 2]. Maxillary lateral incisor is the most common tooth involved in this anomaly [3]. It has been categorized by Oehler into the following 3 types according to the enamel invagination depth [4]: type 1 is limited to the crown, type 2 is extended into the pulp chamber, but doesn’t involve periapical tissues and type 3 is extended to the apical foramina or pseudo apical foramen without any communication with pulp. The type II (as per Oehlers) form exhibits complex internal anatomy and is frequently associated with incomplete root and apex formation [5, 6]. Amongst the three types, types 1 and 2 are considered as incomplete invagination with incidence of 79% and 15%, respectively [6]. 

Conventional radiography are insufficient for diagnosis of the most cases of DIs due to its two dimensional presentation of a three-dimension structure [7]. Thus we used CBCT to observe its extent. CBCT images were obtained in 3 orthogonal planes (axial, sagittal and coronal). CBCT produces three-dimensional images of the anatomy of the teeth and their surrounding tissues with reduced radiation exposure to patient compared to conventional radiography [8]. Several treatment approaches have been described for these anomalies; including non-surgical endodontic therapy, endodontic surgery and finally extraction [3, 9]. This report presents a successful treatment of right maxillary lateral incisor with pulp necrosis and acute per radicular abscess with one-year follow-up by a specific non-surgical endodontic treatment.

## Case Report

A 12-year-old female was referred to endodontic department of Islamic Azad University of Tehran complaining of pain in left maxillary lateral incisor. The patient had no contributory medical and familial history.

**Figure 1:**
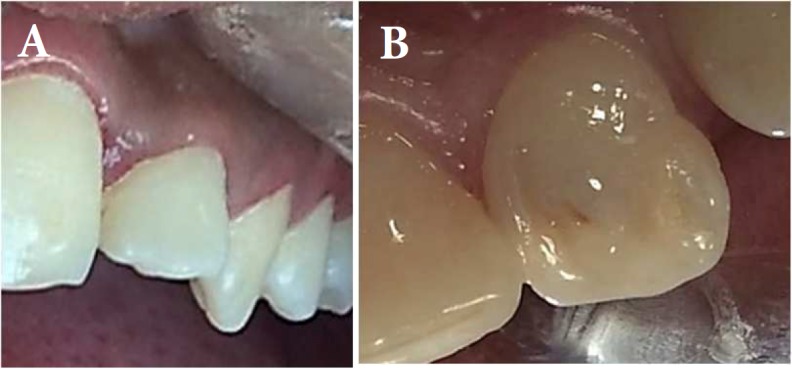
*A)* Labial view; *B)* Palatal view

**Figure 2:**
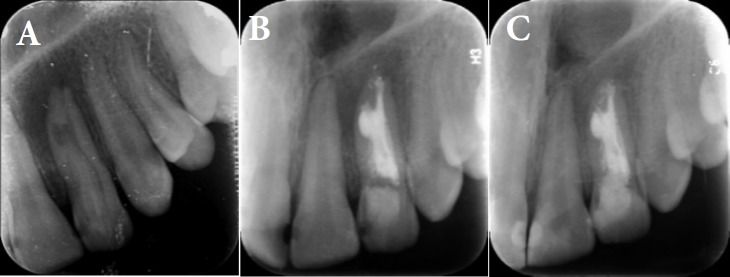
*A)* Preoperative radiography of the maxillary left lateral incisor; *B) *Postoperative radiography immediately after treatment; *C)* Postoperative radiography after one year follow up

**Figure 3:**
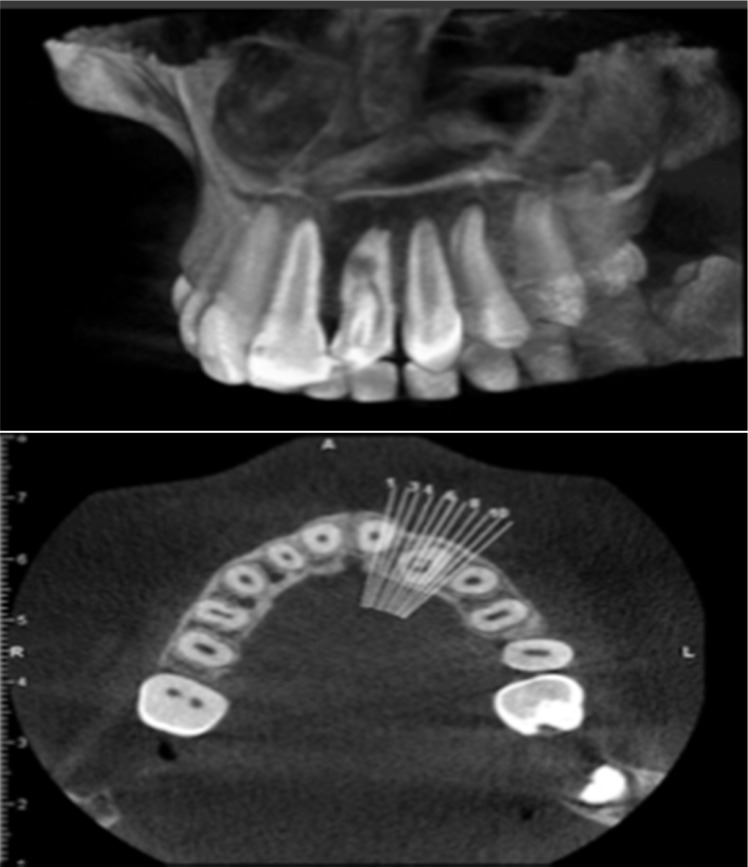
CBCT was used for optimal diagnosis that revealed two separate canals. The invaginated canal was almost at the center of the root but the other canal was located mesiolingually

She did not mention history of pain nor swelling on left anterior of maxilla. She had no dental history. Initial extra-oral examination did not reveal any swelling on the left maxillary lateral. There was a palatal pit in tooth #10 (Figure 1) and the tooth was sensitive to palpation and percussion. An initial periapical radiography and CBCT showed the presence of DI type 2 (Oehler) in tooth #10, associated with a periapical radiolucency and internal root resorption (Figure 2). As the morphology of the invagination was not clear on the periapical radiography, CBCT was used for optimal diagnosis and treatment protocol. The CBCT scan revealed two separate canals. The invaginated canal was almost at the center of the root but the other canal was located mesiolingually. Root resorption associated with invaginated canal was also observed (Figure 3). The patient was treated at two sessions. At the first visit, local anesthesia 2% lidocaine containing 1:80000 epinephrine (Darupakhsh, Tehran, Iran) was applied and the tooth was isolated with rubber dam. The endodontic access cavity was prepared. Then working length was determined by periapical radiography and apex locator. The invaginated canal was found easily and the second canal was found using CBCT. 

Both canals were instrumented using step back technique up to size 50 as the final file. Chemomechanical irrigation was performed with normal saline and passive ultrasonic irrigation with 5.25% NaOCl. Smear layer was removed by 17% EDTA and 5.25% NaOCl. After finishing preparation, the root canals were filled with calcium hydroxide paste and the tooth was temporarily restored by Cavit. After one week in the second visit the temporary coronal restoration was removed and root canals were copiously irrigated by 5.25% NaOCl to remove intra-canal medicament. The main canal was obturated by cold lateral condensation technique with gutta-percha and AH-26 sealer (Dentsplay, Tulsa Dental, Tulsa, OK, USA) and the remaining canal spaces and the internal resorption area was obturated by warm vertical technique (Obtura II, Spartan/Obtura, Fenton, Missouri, USA). The patient was recalled after one year. The tooth had no symptoms and apical lucency was repaired and its size decreased (Figure 2C).

## Discussion

This study reports a difficult case of Oehler’s type II DI in a maxillary left lateral incisor with two separated root canals associated with pulp necrosis and internal root resorption. As CBCT images confirmed, periapical radiographies have limitation in showing type, extension and complex morphology of DI compared to CBCT. CBCT as an advanced device helps in the diagnosis, treatment plan and follow-up with this developmental anatomy [8]. Researchers have suggested a range of alternative methods for treating DI: non-surgical root canal treatment, apexification, surgical procedures or even extraction [2]. Root canal treatment of invaginated tooth is usually challenging due to complex variations of root canal morphology [10, 11].

CBCT is a useful device for the management of complex endodontic problems, because it produces three-dimensional information on the root canal anatomy, teeth and periapical areas with radiation doses lower than the conventional radiography [9]. The attained images from the CBCT data are exclusively useful in evaluating the true nature of the invagination, specially, the relationship of the invagination with the root canal [12]. 

In the presented case no communication was clinically observed between the invagination and oral cavity. Some authors have reported treating the invaginated canal yet retaining pulp vitality in the second canal [3, 13]. CBCT can produce clear three-dimensional images of the tooth without any distortion and has good benefit in the localization and identification of root canals. In this current case, CBCT was helpful in locating the obliterated canal without excessive dentin removal. In the present case the role of CBCT cannot be ignored; it is unseemly that endodontic treatment could have been performed as exactly and safely as it was without the aid of the CBCT images. Because of the irregularities and complex anatomy of the root canal system, in this case, the canals were irrigated plentiful with NaOCl and was medicated with calcium hydroxide. 

## Conclusion

Dens invaginatus is a rare malformation of the teeth, showing a broad spectrum of morphologic variations in size and form of the crowns and roots. Early detection and appropriate preventive measures are of paramount importance in managing these types of dental anomalies. This case report has shown that class II dens invaginatus with an open apex and an apical periodontitis can be successfully treated nonsurgically. CBCT provides more details of internal anatomy of dens invaginatus and is a useful device in the endodontic treatment of this developmental anatomy.
